# Influence of Apical Diameter on Filling Material Extrusion during Retreatment - A Micro-CT and CBCT evaluation

**DOI:** 10.1590/0103-6440202204961

**Published:** 2022-12-05

**Authors:** Thamires Campos Gomes, Jessica de Almeida Coelho, Lucas Rodrigues Pinheiro, Marco Antonio Hungaro Duarte, Patrícia de Almeida Rodrigues

**Affiliations:** 1 Faculdade de Odontologia, Universidade Federal do Pará, Belém, Pará, Brazil.; 2 Faculdade de Odontologia de Bauru, Universidade de São Paulo, Bauru, São Paulo, Brazil.; 3 Centro Universitário do Estado do Pará, Belém, Pará, Brazil

**Keywords:** edema, endodontics, factors associated, pain, root canal therapy

## Abstract

Aim: To investigate whether foraminal widening performed at primary treatment has an effect on the amount of apically extruded obturator material during retreatment and to evaluate the sensitivity of cone beam computed tomography (CBCT) in detecting extruded obturator material. Methods: Forty palatal roots of maxillary molars were selected based on micro-CT and divided into two groups (n=20): with foraminal widening (WE) and without foraminal widening (NE). To standardize the apical foramen, all specimens were instrumented to the foramen using the Protaper Next system, up to instrument X3. The WE group was instrumented to the foramen up to instrument X5, and the NE group was instrumented 1 mm lower. The canals were obturated 1 mm below the apical foramen with gutta-percha and AH Plus and stored for 7 days at 37 °C and 95% humidity. Roots were fixed in microtubes filled with 1.5% agar gel. The obturation material was removed with Reciproc R50. Scans of the teeth and agar were performed using micro-CT and CBCT. Comparison between groups and between methods was performed using Mann-Withney test (p ≤0.05). Results: No statistical difference was found when comparing the extruded material between groups using micro-CT (p = 0.589) or CBCT (p = 0.953). CBCT measured a greater volume of extruded material than micro- CT (p = 0.0004). Conclusion: Foraminal widening had no effect on the extrusion of filling material during retreatment. The CBCT favored the evaluation of apically extruded filling material.

## Resumo

Objetivo: Investigar se o alargamento do foramen realizado durante o tratamento primário tem um efeito na quantidade de material obturador apicalmente extruído durante oretratamento e verificar a sensibilidade da tomografia computorizada de feixe cônico (TCFC) na detecção de material obturador extruído. Métodos: Quarenta raízes palatinas de molares superiores foram selecionadas de acordo com a microtomografia computorizada, e foram divididas em dois grupos (n=20): com alargamento do foraminal (CA) e sem alargamento do foraminal (SA). Para padronizar o forame apical, todas as amostras foram instrumentadas com o sistema ProTaper Next até ao instrumento X3 até o forame. O grupo CA foi instrumentado até ao instrumento X5 até o forame, e o grupo SA foi instrumentado 1 mm aquém. Os canais foram obturados 1 mm abaixo do forame apical com gutta-percha e AH Plus e armazenados durante 7 dias a 37 °C e 95% de umidade. As raízes foram fixadas em microtubos preenchidos com gel de ágar a 1,5%. O material obturador foi removido com Reciproc R50. Os escaneamentos dos dentes e do ágar foram realizados com micro-CT e CBCT. A comparação entre grupos e entre métodos foi realizada utilizando o teste Mann-Withney (p ≤0.05). Resultados: Não foi encontrada diferença estatística ao comparar o material extruído entre grupos utilizando o Micro-CT (p = 0,589) ou TCFC (p = 0,953). Foi medido um volume maior de material extruído com a TCFC do que com a Micro- CT (p = 0,0004). Conclusão: O alargamento foraminal não teve qualquer efeito na extrusão do material obturador durante o retratamento. A TCFC favoreceu a avaliação do material de obturador apicalmente extruído.

## Introduction

Extrusion of filling material into the periapical region is considered one of the causes of failure after retreatment ^1^. Microorganisms may be present in the extrusion of the filling material, creating an imbalance in the host aggression-defense ratio ^1^. The apically extruded gutta-percha has an irregular appearance with edges and peaks that may cause a foreign body reaction or trigger failure after retreatment ^1^. In this way, the host is mobilized in an acute state to restore balance ^2^.

There is clinical and histologic evidence that the presence of foreign bodies that irritate periapical tissues-such as filling materials-impairs the healing of periapical tissues after endodontic treatment ^3^. Host defense cells that accumulate at foreign body reaction sites are important sources of inflammatory and bone resorptive cytokines and other mediators ^3^. In Ricucci, Rôças ^4^, only 15% of cases using AH Plus Sealer showed complete removal of extruded material when assessed after four years.

Studies in the literature have shown that apical enlargement can reduce the bacterial load on the root canal system ^5^. This procedure should prevent contaminated dentin, pulp debris, and microorganisms from interfering with the repair process after endodontic treatment ^6^. In this sense, apical preparation via a #30-diameter instrument would improve decontamination.

Despite the benefits in terms of microorganism reduction, more conservative preparations have been suggested to achieve better preservation of tooth structure and reduce susceptibility to crack formation and canal transport ^7^ ^,^ ^8^ ^,^ ^9^ ^,^ ^10^ ^,^ ^11^.

The most commonly used method to assess apically extruded debris was the weighing method before and after root canal preparation. This method has been challenged by the lack of laboratory conditions that simulate the resistance of periapical tissue to debris extrusion ^12^. Micro-computed tomography (micro-CT) can be used to measure the volume of extruded material with greater precision ^12^. However, this method can not be used in clinical practice. It is necessary to develop an efficient method to evaluate the filling material for clinical use. Thus, cone-beam computed tomography (CBCT) can be a method to clinically detect the extrusion of material into the periapical region ^13^ ^,^ ^14^. Therefore, the aim of the present study was to compare the influence of foraminal enlargement-performed during primary endodontic treatment-on the volume of extruded filling material when endodontic retreatment is required and to evaluate the sensitivity of CBCT in detecting extruded filling material.

## Materials and methods

The local ethics committee reviewed and approved this study (protocol: 3.556.733). The sample was calculated considering data from a previous study ^15^ using teeth with a single canal, with a beta power of 0.80 and a sample number of 34. In this way, taking into account possible losses, 40 straight palatal roots of maxillary molars were selected. The sample was selected based on clinical and radiographic analysis in the mesiodistal and buccopalatal directions. Roots with deviations in root canal volume, the presence of more than one canal, calcifications, flattening or incomplete rhizogenesis were excluded.

### Standardization of the initial apical diameter

To select canals with similar anatomic features and to allow equivalent distribution of specimens into groups according to the volume of the canal expressed in mm³, specimens were digitized on a micro-CT system (SkyScan 1174v2; Bruker-microCT, Kontich, Belgium) at 50 kV, 800 mA, and an isotropic resolution of 19.7 μm ^16^. Images were reconstructed with NRecon software (Bruker) using a 50% beam-hardening correction, a smoothing of 5, and a ring artifact correction of 6.

The structural model index (SMI) values was used to select cylindrical canals. When the SMI was close to 3, it was considered cylindrical ^17^ ^,^ ^18^. The volume of the root canals in the last 12 mm was calculated using CTAn v. 1.12 software (Bruker-microCT), and the normality of the sample was determined using the Shapiro-Wilk test for normality (p = 0.248). In addition, the apical foramen diameter was evaluated and only teeth with similar diameters were selected for the study (from 0.20 to 0.25 mm). Teeth that did not meet this last criterion were excluded.

Forty teeth were then divided into two groups, namely: (a) with foraminal enlargement (WE), n= 20; and (b) without foraminal enlargement (NE), n=20.

Dental crowns were removed, and palatal roots were cut with a double-sided diamond disk (KG Sorensen, São Paulo, Brazil) coupled to a straight piece under constant cooling. All roots were standardized to 12 mm. A Kerr #10 instrument (Dentsply-Maillefer, Petrópolis, Brazil) was used to explore the roots in their entire extension until the tip of the instrument emerged from the apical foramen. The total length of the roots was determined based on this exploration.

All roots were instrumented up to the X3 instrument ^30^ ^,^ ^7^ of the Protaper Next system (Dentsply Maillefer, Ballaigues, Switzerland) in the total length of the canal (12 mm) to standardize an inicial apical diameter.

### Protocols for instrumentation

Instrumentation was then continued to the X5 instrument (50.06) at the working length corresponding to each group (WL). For the WE group, WL was set to 12 mm, while the NE group was set to 11 mm. The canals were rinsed with 5 mL of 2.5% sodium hypochlorite solution after each instrument insertion using a Navitip 30G needle (Ultradent Products, Indaiatuba, Brazil). Each instrument was used only once, and 30 mL of NaOCl was used per canal. At the end of instrumentation, the canals were rinsed with 3 mL of 17% EDTA solution for three minutes, followed by 5 mL of saline.

### Obturation procedure

A single gutta-percha cone with 0.06 taper and 0.50 mm tip diameter was selected for filling. To obtain an equal filling limit of 1 mm below the apical foramen for the two groups, 1 mm of the cone tips of the WE group were cut off with a scalpel blade. The cone selection was confirmed by radiographic examination. The AH Plus cement (Dentsply DeTrey, Konstanz, Germany) was then processed with equal parts of the paste as recommended by the manufacturer, involving the entire primary cone. The gutta-percha cone was cut at the cervical level, and radiographs and CBCT confirmed the quality of the obturation. The cavities were sealed with temporary cement (Maquira Indústria de Produtos Odontológicas S.A., Maringá, Brazil) and the roots were stored in an oven at 37 °C and 95 % relative humidity for seven days to allow the filling cement to finally cure.

Apparatus for the analysis of the extruded material

Following the model proposed by Alves et al. ^12^ a device was made to analyze the amount of extruded filling material. Holes were drilled in the caps of the microtubes (Axigen®, São Paulo, Brazil) to fix the roots. The microtubes were filled with 1.5 mL of 1.5% agar gel (Dinâmica Química Contemporânea Ltda., Diadema, Brazil). The roots were placed in the microtubes, and the root connections were sealed with cyanoacrylate (Loctite Corporation, Duesseldorf, Germany) to prevent leakage of the liquid. The microtubes were coated with blue synthetic nitrile rubber to prevent the operator from observing the retreatment phase. A non-transparent tube support was used to stabilize the device during removal of the filling material.

### Root filling removal technique

**Figure 1:**
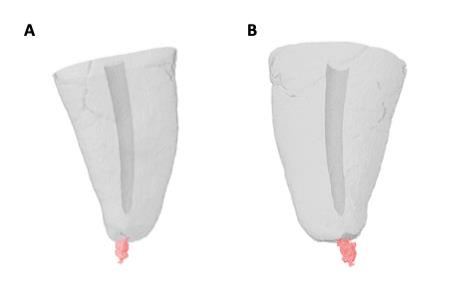
Three-dimensional micro-CT reconstruction exhibiting apically extruded filling material in (A) Teeth without foraminal enlargement and (B) Teeth with no foraminal enlargement.

**Figure 2:**
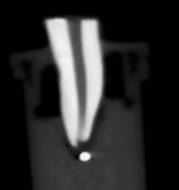
CBCT exhibiting apically extruded filling material.

The filling material was removed with the Reciproc System (VDW GmbH, Munich, Germany), R50 instrument (50.05) driven by an X-Smart Plus motor (Dentsply Maillefer, Ballaigues, Switzerland) in RECIPROC ALL mode. The instrument was guided to the apex with pecking movements with an amplitude of approximately 3 mm. After three movements, the instrument was removed from the canal, cleaned with sterile gauze, and the canal irrigated with 2 mL of 2.5% NaOCl. This procedure was continued until the instrument reached the filling length. Finally, the canal was irrigated with 2 mL of 2.5% NaOCl and apical patency was established with a K #15 file along the entire length of the canal. The material extruded during the removal of the root filling was placed in agar gel, which allowed analysis by imaging micro-CT (Figure 1) and CBCT (Figure 2).

### Micro- CT scan

Roots attached to the device for analysis were digitized in a micro-CT scanner (SkyScan 1174 v. 2; Bruker-microCT, Kontich, Belgium) before instrumentation and after retreatment as described above, at 50 Kv, 800 mA, with a 17 µm pixel, 360º around the vertical axis, a rotation step of 1.0 and 2 frames, using a 0.5-mm-thick aluminum filter. After scanning, the images were reconstructed using the NRecon software, version. 1.6.9 (Bruker-microCT) with a ring artifact correction of 6, a beam hardness correction of 50%, and a smoothing of 5 to create axial and transverse sections of the internal structure of all root canals.

### CBCT scanning

The unit was scanned with a CBCT scanner, PaX-i3D (VATECH Innovative & Reliable Partner, Hwaseong-si, Korea), operating at 92 kVp, 10 mA, a field of view of 5 x 5 cm, and a voxel size of 0.08 mm, before root canal obturation and after root canal retreatment.

### Image analysis

Quantitative analysis of the volume (mm3) of the extruded filling material was performed using CTAn v. 1.5.4.0 software (Bruker-microCT) for both scans. For this purpose, the region of interest of the volume of the residues extruded into the gel was selected and the residues were quantified using a three-dimensional analysis with a morphometry plugin.

### Statistical analysis

The Shapiro-Wilk test was used to assess the normality of the data and was not confirmed. Therefore, the Mann-Whitney test was applied to compare the extruded volume in the WE and NE groups and to make a comparison between the two analytical methods with respect to the measurement of the volume of material in the samples showing extruded material. The significance level set was 5%.

## Results

**Figure 3:**
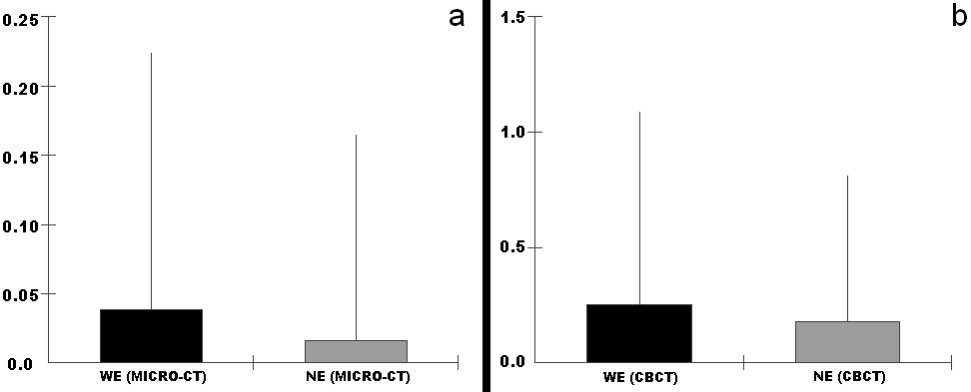
Box plot of the comparison between extrusion (mm³) in the WE group (with foraminal enlargement) and NE group (no foraminal enlargement), assessed by Micro-CT (a) and CBCT (b).

One root from each group was lost during the filling process, leaving a total of 19 roots per group. Of the total samples, seven roots in the WE group and six in the NE group had filling material extruded. The extrusion data are shown in Figure 3. When comparing the extrusion between the groups WE and NE, no statistical difference was found, both when assessed with Micro-CT (p = 0.589) and with CBCT (p = 0.953).

Significant differences were observed in the volumes of extruded material compared between micro-CT and CBCT (p = 0.0004). CBCT showed a higher average volume of extruded material than micro-CT (Table 1).

**Table 1:** Volumetric analysis (mm³) of the extruded material using Micro-CT and CBCT

	Mean (n)	Median	Min-Max
Micro-CT	0.101 (13)	0.074	0.009-0.223^a^
CBCT	0.527 (13)	0.642	0.036-1.086^b^

## Discussion

In both groups, extrusion of filling material occurred after retreatment, thus accepting the null hypothesis that previous foraminal enlargement would not influence the extrusion of filling material (p = 0.5335). These results underscore that regardless of the primary preparation technique, the possibility of filling material extrusion during removal exists. The available clinical evidence suggests that foraminal enlargement provides greater healing in cases with necrotic pulp ^6^. However, since there is no technique that ensures uniform success of endodontic therapy, it is necessary to evaluate the consequences of these techniques in a possible case of retreatment. The null hypothesis of no difference between image analysis techniques was not accepted. CBCT favored overestimation in the evaluation of apically extruded filling material.

In this sense, several techniques for retreatment were investigated, and some degree of filling material extrusion was observed in all of them ^19^ ^,^ ^20^. The extrusion of filling material was mainly studied in terms of the removal protocol, indicating a major influence of the techniques used. The technique using manual instruments is responsible for a greater filling material apical extrusion ^21^ ^,^ ^22^. As for the mechanical systems, some authors have found no difference between the apical extrusion of filling material by rotary or reciprocal systems ^20^ ^,^ ^23^. However, this view is controversial in the literature. This can be justified by the different analytical methods and removal protocols. However, reciprocal systems allow for faster ^24^ ^,^ ^25^ and more effective removal of material ^24^. For this reason, it was decided in this study to use the Reciproc instrument to remove the filling material.

Collecting and weighing the extruded material after the different stages of endodontic treatment is the most important method to quantify these deposits ^20^ ^,^ ^22^ ^,^ ^26^. For this purpose, the material is usually collected using microtubes that are placed in an oven for drying and subsequent weighing. A weakness of this method is that the resistance offered by the periapical tissue is not restored, allowing for a larger extrusion volume. Agar gel (1.5%) was first used by Lu et al. ^27^ to simulate periapical tissues. These authors reported that the density of agar gel could be similar to that of periapical tissue (agar = 1045 kg/m³ and human tissue = 1000-1100 kg/m³). As in the present study, Alves et al ^12^ used a device that simulated the resistance of the periodontal ligament, and this was the first study to use micro- CT images to evaluate the extrusion of debris in primary endodontic treatments.

Extrusion of filling material was noted in 4 (36%), 8 (73%), and 7 (64%) canals in the ProTaper Universal, TruShape, and Reciproc Blue groups, respectively, which also did not represent a statistically significant difference ^28^. These data are greater than those obtained in the present study in relation to the removal of filling material (36% for the WE group and 31% for the NE group). However, the average volume of total material extruded in the present study (0.034 mm³) was greater than the volume of filling material extrusion determined by these authors. Despite the methodological difference between the studies, the comparison of the data draws attention to the volume of contaminated filling material extruded into the periapical tissue during endodontic retreatment. In the present study, the volume of extruded material measured with CBCT was greater than that observed with Micro-CT. Celikten et al ^13^ found larger volumes in root canals evaluated with CBCT compared to those observed with Micro-CT. Decurcio et al ^14^ also observed larger dimensions of root canal filling in CBCT images compared with the original root specimens. These differences may occur due to interference in the imaging systems caused by the different densities of the materials, leading to interpretation errors in CBCT examinations ^29^. When using micro-CT, this condition is controlled by the ability to correct or reduce the size of the devices using image filters in the NRecon software. Despite the statistical difference between the volumes found with the two methods, there was no false-negative result in any of the specimens in the present study, i.e., situations detected with Micro-CT and not observed with CBCT. This shows that CBCT seems to be an interesting tool for clinical use despite its lower accuracy than micro-CT.

Al-Omari et al ^30^ compared the amount of debris extrusion using Reciproc Blue and XP Endo Shaper Systems during retreatment and concluded that filling materials are extruded beyond the apex during this procedure, regardless of the rotary system used. The use of extracted teeth has its limitations. Although agar gel was used to simulate periapical tissue, the results of this study might be different if applied in a clinical situation, since *in vivo* the apex is surrounded by periradicular tissue that may limit apical extrusion. From a clinical perspective, extrusion of filling material may delay periapical healing and cause a foreign body reaction ^1^ ^,^ ^3^. CBCT allow visualization of the filling material outside the canal, unlike the other methods that weigh the extruded material ^12^. However, further studies should be conducted to assess the extent to which apical extrusion of filling material may affect the success of endodontic treatment, considering that this study measured the volume of extruded material and whether it can be detected by CBCT, but the clinical effects of filling material extrusion were not evaluated.

In conclusion, foraminal enlargement performed during primary endodontic treatment was not a factor influencing the extrusion of filling material into the periapical region during endodontic retreatment. CBCT is a suitable tool to detect the extruded filling^)^ material.
